# *Klebsiella pneumoniae* sepsis complicated with central nervous system involvement: A case series

**DOI:** 10.1002/ccr3.6793

**Published:** 2022-12-22

**Authors:** Ken Inoue, Mayu Hikone, Masato Oishio, Saki Noji, Kazuhiro Sugiyama

**Affiliations:** ^1^ Tertiary Emergency Medical Center (Trauma and Critical Center) Tokyo Metropolitan Bokutoh Hospital Tokyo Japan

**Keywords:** central nervous system, sepsis, multiple organ failure

## Abstract

Central nervous system (CNS) infection with *Klebsiella pneumoniae* can be a complication of invasive liver abscess syndrome; however, CNS infections due to *K. pneumoniae* without liver abscesses are rare. We report three fatal cases of CNS infection due to *K. pneumoniae* that lacked liver abscesses during the initial investigation.

## Graphical abstract

*Klebsiella pneumoniae*‐related sepsis may be associated with CNS infection even without liver abscesses in initial imaging studies. *Klebsiella pneumoniae* should be recognized as a causative pathogen that may complicate CNS infections with a poor prognosis.

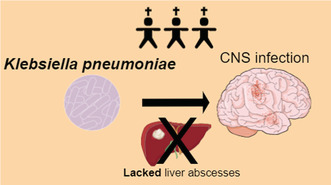

## 1 INTRODUCTION

*Klebsiella pneumoniae* (*K. pneumoniae*) causes nosocomial and community‐acquired infections of the urinary and biliary tracts and pneumonia. Community‐acquired central nervous system (CNS) infections with *K. pneumoniae* are rare among adults in most countries,^1^, ^2^, ^3^, ^4^, ^5^ including Japan.^4^, ^5^ Invasive liver abscess syndrome (ILAS) due to *K. pneumoniae* is known to cause CNS metastatic complications, resulting in high morbidity and mortality rates.^6^, ^7^ ILAS emergence may contribute to an increase in *K. pneumoniae* sepsis complicated with CNS infections. The presence of a liver abscess is an important clinical clue when suspecting this fatal disease. When there is no liver abscess observed on initial investigation, *K. pneumoniae* may not be considered as a differential. Taiwan is experiencing a higher incidence of community‐acquired adult bacterial meningitis due to *K. pneumoniae*.^8^, ^9^ Other countries, however, may not be following this trend and may seldom encounter *K. pneumoniae‐*related CNS infections in community settings.

We report three cases of *K. pneumoniae* sepsis complicated with CNS infection that lacked findings of liver abscess during the initial investigation. This case series aimed to raise awareness among clinicians that *K. pneumoniae*‐related sepsis can complicate CNS infections and to prompt clinicians to promote rapid and appropriate investigation and therapeutic strategies as this disease can be fatal.

## 2 CASE PRESENTATIONS

### 2.1 Case 1

**FIGURE 1:**
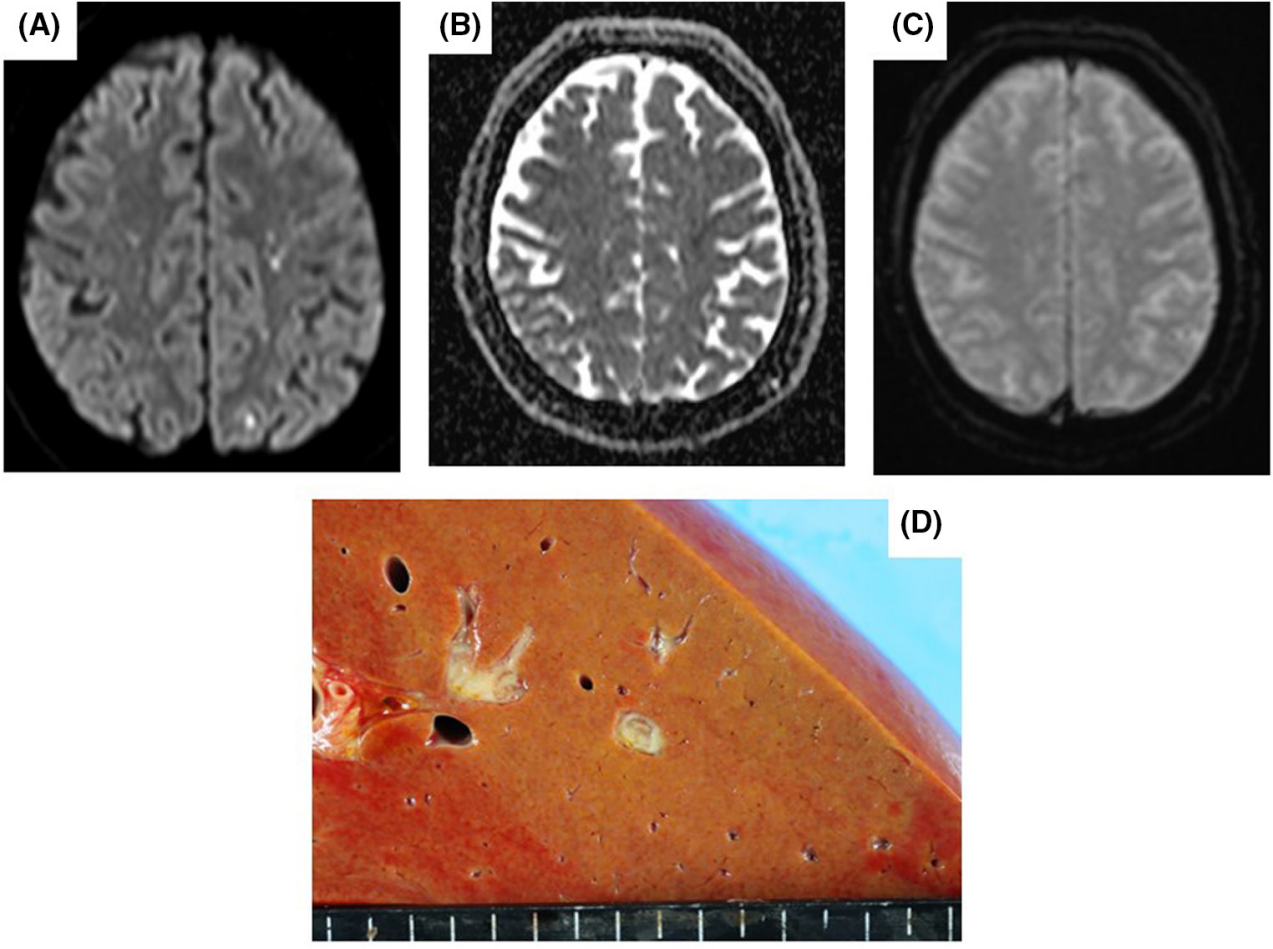
(A–C) Magnetic resonance imaging scans in Case 1 showing multiple foci of restricted diffusion in the cortimomedullary junction. (A) The diffusion‐weighted image. (B) The apparent diffusion coefficient image. (C) The T1‐weighted image. (D) Autopsy investigation findings in Case 1 showing a small abscess in the liver.

A 63‐year‐old man with diabetes mellitus, hypertension, and no history of neurosurgery was transferred to our facility with a 2‐day history of worsening consciousness disturbance, fever, and vomiting. Initial examination revealed a Glasgow Coma Scale (GCS) score of 3 (E1V1M1) with anisocoria; body temperature (BT), 36.7°C; pulse rate (PR), 150 beats/min; respiratory rate (RR), 40 breaths/min; blood pressure (BP), 109/73 mmHg; and oxygen saturation (SPO_2_), 100% on 10 L/min of oxygen via a reservoir mask. Laboratory results showed elevated inflammatory marker, creatinine, and liver enzyme levels; thrombocytopenia; and lactic acidosis. Computed tomography (CT) with contrast showed septic emboli in both lungs; otherwise, the infection focus was not observed. Diffusion‐weighted magnetic resonance imaging (DW‐MRI) showed disseminated hyperintensities in the brain, possibly due to infectious embolisms or micro‐abscesses. The observed pattern was consistent with embolic lesions (Figure 1A–C). Under the clinical diagnosis of septic shock complicated with pulmonary and CNS dissemination, fluid resuscitation, vasopressor support, steroid and antibiotic administration, and mechanical ventilation were initiated, and he was admitted to the intensive care unit (ICU).

Shortly after admission, his hemodynamic state deteriorated rapidly, leading to cardiac arrest, which required veno‐arterial extracorporeal membrane oxygenation for resuscitation. Despite aggressive treatment, the patient died 4 days after admission. Blood cultures indicated *K. pneumoniae* with a positive string test result. Autopsy revealed turbid cerebrospinal fluid and inflammatory cell infiltration in the spinal cord, consistent with bacterial meningitis. A liver abscess was also detected, which was not apparent on the initial CT scan (Figure 1D). Based on his clinical course and autopsy, the patient was diagnosed with *K. pneumoniae* sepsis with liver abscess and meningitis.

### 2.2 Case 2

A 63‐year‐old man with diabetes mellitus and no history of neurosurgery was transferred to our facility because of consciousness disturbance and headache beginning 1 day prior. Initial examination results showed a GCS score of 8 (E1V2M5); BT, 39.0°C; PR, 171 beats/min; RR, 31 breaths/min; BP, 103/71 mmHg; and SPO_2_, 99% on 6 L/min of oxygen; and a stiff neck. Laboratory results showed a decreased white blood cell count, thrombocytopenia, elevated creatinine and liver enzyme levels, coagulopathy, and lactic acidosis. CT scans showed a dirty fat sign around the prostate and no space‐occupying lesions in the brain or liver. The cerebrospinal fluid showed leukocytosis (10,200/3 μl) with abundant polynuclear leukocytes (5760/3 μl) and an elevated protein level (2122 mg/dl), consistent with bacterial meningitis. Under the clinical diagnosis of septic shock complicated with prostatitis and meningitis, fluid resuscitation, vasopressor support, antibiotic administration, and mechanical ventilation were initiated, and he was admitted to the ICU.

Despite intensive care, his hemodynamics deteriorated, and he died 8 days after admission. Blood and cerebrospinal fluid cultures were positive for *K. pneumoniae* (string test not performed). Autopsy findings indicated a prostate abscess but no liver abscess. Based on the autopsy, he was diagnosed with *K. pneumoniae* sepsis with prostate abscess and meningitis.

### 2.3 Case 3

A 73‐year‐old man with schizophrenia and no history of neurosurgery was transferred to our facility because of consciousness disturbance. Initial examination findings indicated a GCS score of 11 (E4V2M5); BT, 38.2°C; PR, 121 beats/min; RR, 30 breaths/min; BP, 121/71 mmHg; and SPO_2_, 97% on 10 L/min of oxygen. Laboratory test results showed elevated inflammatory marker, creatinine, and liver enzyme levels; thrombocytopenia; coagulopathy; and lactic acidosis. CT scans showed fluid retention around the gallbladder and small liver cysts without ring enhancement. His head CT was unremarkable. Under the initial clinical diagnosis of sepsis of unknown origin, fluid resuscitation, vasopressor support, antibiotic administration, and mechanical ventilation were initiated, and he was admitted to the ICU.

**FIGURE 2:**
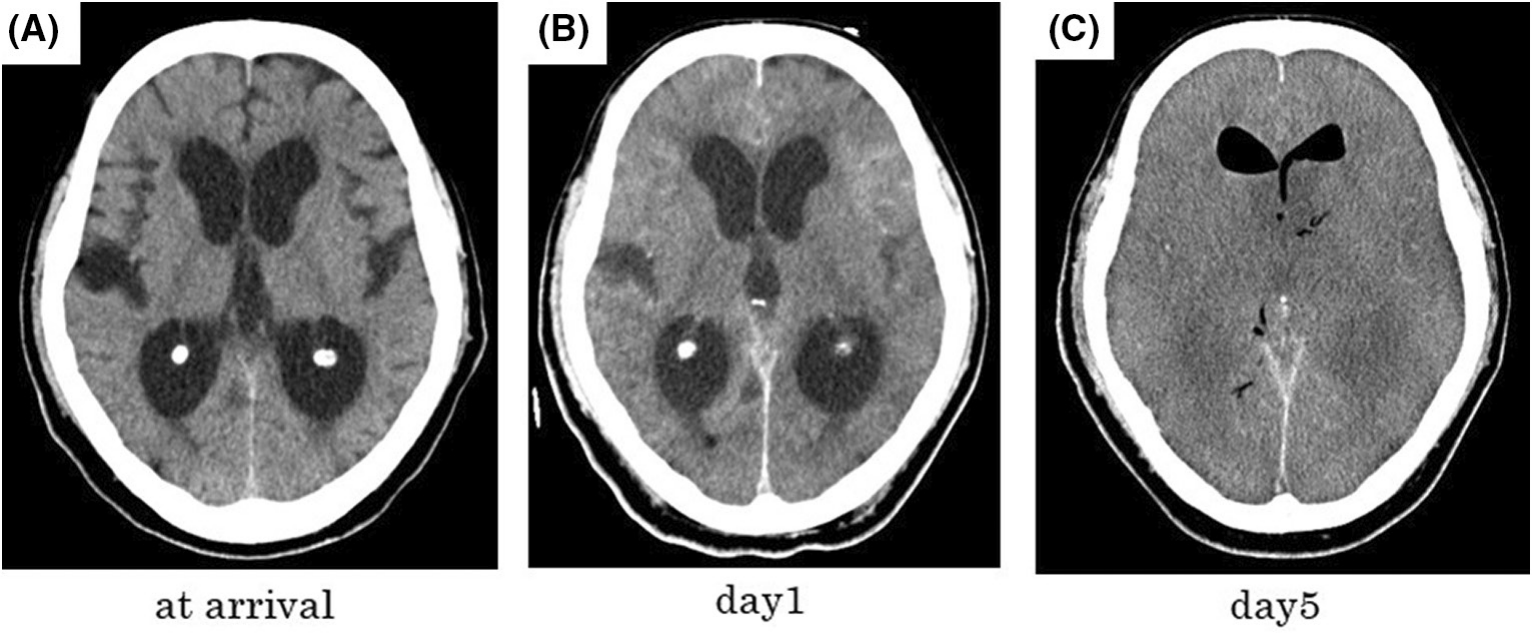
(A‐C) Head computed tomography scans in Case 3 showing diffuse sulcal effacement over time and air replacement in the ventricles. (A) at arrival (B) on day1 of hospitalization (C) on day5

Since bilateral dilated pupils and disappearance of the light reflex were newly noted after admission, a follow‐up head CT scan was performed, which showed cerebral edema, a high‐density area within the subarachnoid space, and an obscured corticomedullary junction (Figure 2B). The cerebrospinal fluid indicated leukocytosis (50,133/3 μl) with abundant polynuclear leukocytes (46,400/3 μl), an elevated protein level (1020 mg/dl), and a low glucose level (4 mg/dl), consistent with bacterial meningitis. A subsequent CT scan performed on Day 5 showed air in the ventricle and worsening cerebral edema (Figure 2C). The patient died 6 days after admission. Blood culture results were positive for *K. pneumoniae* with a negative string test result. He was diagnosed with *K. pneumoniae* sepsis with meningoencephalitis.

## 3 DISCUSSION

*Klebsiella pneumoniae* rarely causes community‐acquired CNS infections in most countries, including Japan.^1^, ^2^, ^3^, ^4^, ^5^ Meningitis due to gram‐negative bacilli, including *K. pneumoniae*, is uncommon, with only a few cases having been described in a multicenter survey and in a hospital‐based survey undertaken in Japan.^4^, ^5^ This trend is similar in the United States^1^ and in Europe^2^, ^3^ whereas, in Taiwan, the incidence of community‐acquired bacterial meningitis due to *K. pneumoniae* has been reported to be high.^8^, ^9^ This epidemiological difference may be related to the accumulation of cases of hypervirulent *K. pneumoniae* (*hvKp*) in certain Asian countries. A feature of hv*Kp* is hypermucoviscosity, which is conventionally identified using a positive string test,^10^ and other microbiological and genetic markers are known to characterize hypervirulent strains of *K. pneumoniae*. *HvKp* causes an invasive and fatal syndrome with community‐acquired primary liver abscesses complicated with disseminated lesions, now widely referred to as ILAS. Disseminated lesions often include CNS manifestations, leading to a poor clinical course.^6^, ^7^ Cases of ILAS originating from Asian countries have now been reported globally, and ILAS is regarded as an emerging infectious disease. Several underlying diseases, such as diabetes mellitus and liver disease, especially cirrhosis, malignancy, and heavy alcohol consumption, are considered risk factors,^7^, ^9^, ^11^ although cases of ILAS have also been noted in healthy individuals.^6^

Most cases of community‐acquired *K. pneumoniae* sepsis complicated with CNS infection manifest as ILAS, and cases without liver abscess or liver abscess not being apparent are rare. Here, no liver abscess findings were observed on the initial CT scan or autopsy in Cases 2 and 3, whereas liver abscess at autopsy was observed in Case 1 but not detected during the initial CT scan. In Case 2, a prostate abscess was identified during autopsy. *K. pneumoniae* can produce primary abscesses in non‐liver organs and cause disseminated infections. Our cases indicate that *K. pneumoniae* sepsis with CNS complications, which resembles the clinical picture of ILAS, may not always present with evident liver abscesses.

Magnetic resonance imaging is a useful method for differentiating consciousness disturbance. Bacterial meningitis can form subdural empyema, which may show as hyperintensity on DW‐MRI, as hypointensity on a restricted apparent diffusion coefficient, and as ring enhancement on T1‐weighted images.^12^, ^13^ In Case 1, brain images were consistent with infectious change.

In these three cases, the clinical courses showed invasive *K. pneumoniae* infection with CNS involvement; however, it is important to note that microbiological investigation was limited. Only Case 1 showed a positive string test whereas, in Cases 2 and 3, microbiological information was either not sought or testing was negative, respectively, in relation to the characteristics of hypervirulent strains. Detailed microbiological investigations in future similar cases are likely to help enhance understanding of this fatal disease.

Management of these invasive and rapidly deteriorating infections requires prompt initiation of antimicrobial therapy with a CNS infection dose and intensive care to support the hemodynamic state. While drainage of liver abscess in cases of ILAS has been associated with improvement in mortality, metastatic infections, and complications,^14^ drainage may not be considered when a primary liver abscess is not apparent. Further research is warranted to identify risk factors and effective treatment strategies, as this infection requires early recognition and therapeutic intervention to improve prognosis.

## AUTHOR CONTRIBUTIONS

**Ken Inoue:** Conceptualization; project administration; writing – original draft. **Mayu Hikone:** Supervision; writing – review and editing. **Masato Oishio:** Writing – review and editing. **Saki Noji:** Writing – review and editing. **Kazuhiro Sugiyama:** Supervision.

## FUNDING INFORMATION

None.

## CONFLICT OF INTEREST

None.

## ETHICAL APPROVAL

Not applicable.

## CONSENT

The patients provided consent for publication permission to reproduce material from other sources.

## Data Availability

The data that support the findings from this study are available from the corresponding author upon reasonable request.
